# “The Notion of Neutrality in Clinical Ethics Consultation”

**DOI:** 10.1186/s13010-018-0056-1

**Published:** 2018-02-27

**Authors:** Alessandra Gasparetto, Ralf J. Jox, Mario Picozzi

**Affiliations:** 1 Center for Clinical Ethics, Biotechnology and Life Sciences Department, Insubria University, Varese, Italy; 2 Institute of Ethics, History and Theory of Medicine, Ludwig-Maximilian University (LMU), Munich, Germany

**Keywords:** Clinical ethics, Neutrality, Professionalism, Role, Ethics expertise, Decision-making

## Abstract

Clinical ethics consultation (CEC), as an activity that may be provided by clinical ethics committees and consultants, is nowadays a well-established practice in North America. Although it has been increasingly implemented in Europe and elsewhere, no agreement can be found among scholars and practitioners on the appropriate role or approach the consultant should play when ethically problematic cases involving conflicts and uncertainties come up. In particular, there is no consensus on the acceptability of consultants making recommendations, offering moral advice upon request, and expressing personal opinions. We translate these issues into the question of whether the consultant should be neutral when performing an ethics consultation. We argue that the notion of neutrality 1) functions as a hermeneutical key to review the history of CEC as a whole; 2) may be enlightened by a precise assessment of the nature and goals of CEC; 3) refers to the normative dimension of CEC. Here, we distinguish four different meanings of neutrality: a neutral stance toward the parties involved in clinical decision making, toward the arguments offered to frame the discussion, toward the values and norms involved in the case, and toward the outcome of decision making, that is to say the final decision and action that will be implemented. Lastly, we suggest a non-authoritarian way to intend the term “recommendation” in the context of clinical ethics consultation.

## Background

The question of neutrality in clinical ethics consultation (CEC) has accompanied the international debate from its very beginning, even though it has rarely been explicitly addressed. This question has existed since the origin of clinical ethics and has persisted with its developing presence in the clinical sphere through the formal establishment of clinical ethics committees. Initially, clinical ethics committees were endorsed by the New Jersey Supreme Court in the context of Karen Quinlan’s case (1976), later by the President’s Commission for the Study of Ethical Problems in Medicine and Biomedical and Behavioral Research (1983), as well as by several other public and professional bodies. According to the President’s Commission in 1983, the committees were supposed to perform ethical case review offering “non-binding advisory recommendation” ([1], p. 440). While committees have been primarily created to support the decision-making process of healthcare professionals, the task of individual consultation has always been the most controversial task of clinical ethics committees. Some scholars wanted to frame CEC in a more modest way as simply a discussion, while others even argued in favor of stopping individual consultation altogether and focusing on education and policy development.

Clinicians often voiced concerns about the supposed moral authority of a committee consisting of various experts. Any kind of ethics case consultation carried out by persons external to the clinical team was sometimes regarded as a risky intrusion that could jeopardize the close doctor-patient dyad and undermine the decision-making authority as well as the responsibility of the health care professionals [2]. In fact, according to Fleetwood et al., the ethics committee was expected to function in a way consistent with a clinical consultation model and to provide moral advice, recommendations or “right answers” [3].

These high expectations have become more and more controversial as the limits of the full-committee approach to CEC have led to the gradual appearance of small committee units and single ethics consultants acting directly at the bedside. The skepticism about the consulting activity was evidenced, from the very beginning, by the vague expressions used to designate it. As an example, CEC was referred to using several words, such as “consultation”, “case review”, “counseling”, or “case discussion” ([4], p. 22). Except for “consultation”, which has a clear meaning, i.e. providing advice, the other terms suggest a less demanding task which in no way indicates the right thing to do. We want to show that there are good philosophical reasons to believe that the ethical deliberation, which may even include advice or suggestions, is different from the final decision or action.

### Different models of ethics consultation

It has been claimed that there are at least two big categories under which ethics consultation may be classified, namely the so called “soft” and “hard” models for ethics consultation [5]. These two categories differently conceptualize the extent to which the ethics consultant should be involved in decision-making regarding patient care. Generally speaking, in the first category the ethics consultant may act as a facilitator or a mediator, facilitating the discussion among the involved actors, clarifying their moral positions, exploring options and mediating conflict to reach a solution. The second category, to the contrary, resembles more the clinical model of consultation, and the ethics consultant acts as a clinical professional who possesses a specific expertise and makes substantive recommendations on the ethically best course of treatment. It has been pointed out that the main difference between the two models is the presence of a rendered recommendation [6].

There is no consensus on how acceptable it is for the consultant to provide substantive recommendations regarding patient care. Reviewing the two editions of the American Society for Bioethics and the Humanities (ASBH) reports on Core Competencies for Healthcare Ethics Consultation, there are at least some claims that must be taken into consideration [7, 8]. In the first edition of the report (1998), after having outlined a clinical case in which more than one option was considered ethically feasible, the authors permitted consultants to express personal moral judgements, but expected them to justify and designate them as personal opinions. In the second edition of the report (2011), consultants are cautioned about recommending a particular course of action when more than one is ethically justified; according to the authors, this would qualify as unacceptably authoritarian. Yet, the authors also state that when the parties contemplate a certain decision or action that is clearly regarded as unethical by the consultant, the latter should recommend against it. When just one of the identified courses of action is ethically appropriate, she should state why others are not. Besides, the consultant is allowed to provide “process” recommendations, e.g. she can suggest involving more people in the discussion, or point out that the patient’s decisional capacity needs to be assessed. In any case, both the first and the second edition state that the consultant is committed to helping the parties identify a range of ethically permissible options.

Many scholars and practitioners in the field have dismissed the practice of expressing recommendations, limiting the role of the consultant to the identification of possible options. Once the options are identified as morally acceptable, so they contend, it is not the consultant’s business to tell the parties what the right action is or how they should decide [9].

It has also been argued that specific ethically troublesome cases require the consultant to play a more active and substantial role to help resolve conflicts and dilemmas. The cases defined as “unsettled”, i.e. cases for which clear ethical and professional standards of resolution cannot be found, seem to allow the consultant to go beyond the strictly detached role of a neutral mediator [10]. When it is not possible to identify unambiguous points of reference to assess whether an option is ethically acceptable, the consultant may explore what is ethically permissible, but in the end she is not allowed to recommend it [11, 12]. Others argue for a flexible role of the consultant, moving between neutral moderation, analysis, clear advice and even advocating or insisting on certain normative aspects [13]. Health care professionals might be more or less willing to take advice from the ethics consultant and sometimes clearly ask for such guidance in order to make better informed decisions [14, 15].

Empirical data show that the practice of issuing recommendations is quite common among CEC services in the United States [16]. However, it seems that there is no clear and unambiguous understanding of the term “recommendation”: it may indicate either telling the parties what should be put into practice or just uncovering feasible options ([8], p. 38, note 89). In short, such heterogeneity in theory and practice requires further investigation into the normative dimension of ethics consultation as well as a clear understanding of the role that the consultant is expected to play [16].

In any case, to tell persons who are asking for an ethics consult what should or should not be done is considered unjustified for reasons directly referring to the issue of ethics expertise. Ethics expertise raises both conceptual and moral concerns [17]. Firstly, expertise in ethics is often regarded as a fiction by those who think that there is no real, certain and unequivocal knowledge attainable in ethics. Indeed, normative ethics is an endeavor that cannot be based solely on empirical evidence or fundamental moral claims that are universally accepted. Since ethics is not a discipline that provides objective knowledge, this makes any claim to the usual form of expertise simply inconsistent. The idea of ethics expertise raises criticisms even from a moral perspective: the belief that someone has a better insight into right and wrong seems inconsistent with contemporary moral pluralism and with the tenet of autonomy in decision-making. People may hold well-justified but divergent personal opinions about what is right and what is wrong and no one is in a privileged position as to be able to adjudicate on these opinions. For the reasons mentioned above, several authors favor a cautious role for the ethics consultant so as not to risk authoritarianism and in order to be respectful of today’s moral pluralism. Hence, facilitation and mediation have often been endorsed as the best approaches to ethics consultation [18].

### Ethics expertise

The issue of expertise in ethics would deserve a profound discussion in a separate article. As Lisa Rasmussen rightly summarizes, “Not only do authors disagree on whether ethics expertise exists, they disagree on what it *is*” ([19], p. 2). Here, we can only present some thoughts about ethics expertise for clinical ethics consultants, distinguishing between three levels of expertise.

We consider the clinical ethics consultant as an expert, even though we assume there are at least three main senses in which ethics expertise may be intended and these three senses are subject to growing moral complexity. The first, which we would call the “unproblematic” meaning of ethics expertise, consists in a good knowledge of ethical theories, concepts, principles and arguments, relevant bioethics and clinical ethics literature, guidelines, professional standards of practice, and a basic knowledge of the relevant law. The second, which we would call normative expertise, is more controversial and has to do with the ability to analyze ethical quandaries, find plausible solutions and justify with convincing arguments why a proposed solution is morally good or at least better than the alternatives. This is the meaning of ethics expertise which raises the most criticism, particularly if one takes into account the disagreement on the meta-ethical level, the lack of a unanimous definition of right and good, and the obvious reality of a highly pluralistic society [17].

The third meaning of ethics expertise is even more controversial and refers to practical wisdom as a virtue. Including also the evaluative dimension of how to lead a good life. This expertise does not express a mere cognitive knowledge of the good, but a kind of identification of the self with the knowledge possessed: this practical wisdom implies acting virtuously as second nature. Whether this last meaning is applicable to the ethics consultant would require further in-depth reflection and strong justification. It has already been noted how controversial the use of “character” and “virtue” is in the ethics consultant’s description, considering the lack of a shared definition of the good in our contemporary society [20]. However, as Sidney Callahan put it, a certainly relevant question for ethics consultation is: “Must you be morally wise and good to do bioethics well?” ([21], p. 24).

Within the limits of this contribution we argue for the first and second meaning of ethics expertise. In our understanding, the consultant should possess specific knowledge that we identify with what Stephen Wear calls the “canon of clinical ethics” [22]. That is a set of shared principles, rules, norms and guidelines that should govern the activity of ethics consultation and define its boundaries. Moreover, the consultant should also address and attempt to answer the core question of normative ethics in clinical settings, namely, what the best justified decision is in a specific situation for a particular patient [15].

### Clinical ethics as a normative activity

We argue that the interpretation of the role of the ethics consultant as a simple facilitator or mediator leaves it quite impoverished. Even though it has been largely explained that mediation pays great attention both to “good process” and to “good outcomes”, namely, outcomes consistent with ethical and legal standards, we suggest that clinical ethics has to adopt a normative role for the consultant. In other words, we argue that the clinical ethics consultant has to play a normative enriched role and not just a role of mediation. At the same time, this forces us to answer the question whether the consultant should be neutral. We conceive the clinical ethics consultant not as a neutral mediator or facilitator, but as a real health care professional with her own moral responsibilities. In doing so we defend the thesis that the definition of clinical ethics has inherent normative content with relation to the role of the ethics consultant. Furthermore, we support a non-authoritarian interpretation of the term “recommendation”, which is better understood as “advice”, that is to say, the appropriate involvement or attentive participation of the ethics consultant in the human, ethical and psychological dimension of the case discussed.

For our purposes it is helpful to compare the goals of medicine with the goals of clinical ethics. Clinical ethics shares with medicine a morally-oriented nature aimed at fostering the good of the patient as well as at improving health care assistance. Clinical ethics focuses on good ethical decision-making which should lead to medically and morally appropriate care outcomes [23, 24]. Therefore, there is a clear parallel and even a necessary connection between the goals of medical care and the goals of ethics consultation. By acknowledging that the clinical ethics consultant has professional attitudes and moral responsibilities that are comparable to those of physicians and other health care professionals, we have already partially rejected the notion of neutrality as the feature that should guide the activity of the ethics consultant. If speaking about ethics consultation necessitates reflecting upon the values and norms that should inform and guide the ethically best decision in patient care, then dealing with the question of neutrality means to address the question of normativity in CEC. We will distinguish four different specifications of the term neutrality when it is applied to the process of ethics consultation. In particular, we analyze what neutrality means with regard to (a) the parties involved in the consultation process, including the ethics consultant herself; (b) the arguments offered by the consultant to frame the discussion; (c) the values and norms involved in the case at hand; and (d) the outcome, that is to say the final decision or action that will be implemented. This distinction reflects in part the analysis of “impartiality” in bioethics mediation and ethics consultation made by David Perlman [25].

(a) With regard to the parties participating in the ethics discussion, neutrality is conceived as “impartiality” [25] or “fairness”. This means behaving in a respectful way towards the various interests, preferences and values of the participants, giving them the chance to be equally heard and considered. According to this philosophy, each point of view deserves respect and moral consideration. This is well explained by the ASBH when they state that, in order to “facilitate the building of a principled ethical resolution, the ethics consultant must ensure that involved parties (e.g., patients, families, surrogates, healthcare professionals) have their voices heard” ([8], p. 8). Nevertheless, assuming a clinical consultation model for ethics consultation means to us that the consultant’s primary responsibility is to protect and help realize the good of the patient. Put another way, the consultant should ascertain whether the parties involved (physicians, nurses, relatives) are really acting for the good of the patient or whether, on the contrary, they are pursuing personal interests.

Concerning the notion of neutrality when applied to the person of the ethics consultant, we emphasize that the clinical ethics consultant is as much a moral agent as anyone else involved in the consultation. According to the Code of Ethics for Health Care Ethics Consultants, consultants are cautioned about potential conflicts between personal and professional integrity [26]. Personal integrity is defined as acting in a manner consistent with personal moral values, whereas professional integrity means acting in a manner consistent with professional values aligned with shared ethical standards. When consultants personally have moral objections against certain practices, such as artificial reproductive technologies, they should try to refer the case to a colleague. If this is not possible and there is a clear conflict of interest between personal integrity and professional integrity, the Code argues that professional integrity should be preserved.

As Walter Edinger rightly pointed out, there may be cases in which consultants might feel morally obligated to speak up, feeling that it would be unsatisfactory just to tell parties the prevailing consensus position and to hide behind a veil of silence with respect to their moral positions. Sometimes the consultant’s opinion can diverge from the consensus position. In such cases, giving also one’s own opinion would mean to behave with “moral responsibility” and “conviction” ([27], p. 26). In other circumstances, there may be several ethically well-founded courses of action. In such cases, the consultant may enrich the deliberation by explaining her personal opinion and making it available for further discussion. Of course the consultant, as the other parties, should pursue an unselfish interest in the consultation: she should act for the patient’s good.

(b) The second meaning of neutrality is “objectivity” or “transparency” in the arguments that the consultant may offer. It refers to the ability to offer well-built justifications for ethical claims, to provide rigorous reasons and conclusive arguments in light of ethical standards, institutional policies, literature, precedent cases and empirical evidence. Before being “objective” and “transparent” in relation to the arguments, the consultant must be competent and well-informed. We apply “objectivity” and “transparency” also to the consultant’s personal moral point of view: as already stated, we believe that the consultant is allowed (and sometimes even required) to disclose her moral convictions making sure to underline that they are her own.

The third (c) and the fourth (d) meanings of neutrality are closely linked. When applied to the values and norms involved in an ethics consultation and to outcomes of the consultation, we reject neutrality if it is conceived of as moral indifference. Moral indifference follows from a meta-ethical position which denies that human reason is equipped to find any substantial and shared indication about what is morally good or ethically obligatory. If there is no possibility for human reason to grasp any certain normative content that inspires moral conduct, there is no yardstick for assessing good intentions, actions, and outcomes. This meta-ethical topic cannot be fully covered here and falls outside the scope of this contribution. However, it is sufficient to recall that even though the ASBH reports on Core Competencies do not (and maybe cannot) enter the philosophical question about the sources of human morality, they abundantly adopt expressions like “morally/ethically acceptable”, which generally refer to shared ethical, social and legal standards. Even if there is no clear official standard or definition on what is “ethical”, i.e. “morally good”, the possibility of realizing good outcomes or a “principled ethical resolution” (as it is called in the second edition) is never rejected. As Perlman states, “the ethics consultants cannot be impartial to the outcome or to the norms used to reach that outcome” [25]. In the ASBH reports we recognize a reference to basic common moral rules or criteria that should orient the ethical deliberation towards good choices. This is true even if it is acknowledged that moral pluralism governs the moral life of individuals, now more than ever.

To sum up, in ethics consultation we do not regard as morally indifferent which precise norms or principles are worth protecting and promoting. Sometimes what is needed is a complex moral assessment of the goodness of actions, ends, values and principles considered in the ethical deliberation. Of course this may be done according to some meta-ethical criteria, such as a moral perspective that considers the character of the moral agent, the nature of the moral act, its consequences and the related contextual factors [28]. This is the main contribution the consultant may bring to good ethical outcomes in patient care.

### The moral value of advising

We think there is a difference between the process of “ethical deliberation”, which comes beforehand (and may even include recommendations or advice), and the clinical decision or action, which follows and may be the result of the ethical deliberation. To recommend does not mean to order, to decide, to act, to make moral decisions on behalf of others or to impose moral beliefs. To recommend actions, options or solutions in CEC has largely been misunderstood as undermining the authority of the morally appropriate decision makers or threatening today’s moral pluralism. We would like to rehabilitate the idea of recommendation and to put aside the most controversial aspects of its definition. The term “recommendation” is largely adopted in the clinical sphere by physicians who are acting as clinical consultants. They are right to “recommend” because they are supposed to know what is right to do to restore health and to recover from illness. It raises suspicion when it is used in the field of CEC, because in ethics there is no comparable scientific evidence to prove the superiority of certain recommendations over others. Since the term “recommendation” has the unfortunate connotation of imposing one’s own view on others, we think rather that “advice” has the merit of invoking a partnership in the ethical deliberation [29].

The term “advice” corresponds to the ancient Greek word “symboule”, which means “deliberation” (*boule*) “with” or “together” (*sym*). As Antonio Da Re points out [29], in the Book III of the Nicomachean Ethics (1112b10–11) Aristotle wrote: “We call in others to aid us in deliberation on important questions, distrusting ourselves as not being equal to deciding” ([30], p. 1756). In the original Greek version, Aristotle uses the term “symboulous” for people (i.e. counselors, advisors) who may help us in deciding on important questions when we think we are not able to discern it well enough ourselves [29]. Here, the advisor is a sort of partner in the deliberation process and helps to realize a joint deliberation (“deliberating with or together”). We think this might offer an interpretation of the role of the ethics consultant that highlights her expertise as someone trustworthy and helpful when decisions have to be taken and the decision-makers are not certain about what is ethically the most appropriate action or solution.

We argue that the ethics consultant is allowed to express her personal moral opinion as stated previously. There may be circumstances in which more than one ethically justifiable option is available in a given case, so that the consultant may want to expand the discussion offering her own insight; in other circumstances, she may even disagree with the consensus view [27], and then it is a matter of integrity to give voice to one’s own opinion. However, the consultant should pay attention to clearly state that the opinion is her own. In other situations, the appropriate role of the consultant may be just to clarify and analyze ethical issues or facilitate understanding and agreement among persons. Therefore, it often depends on what the consultant is asked to do or what the situation requires.

We do not contend that the consultant has to know what is good for the patient in absolute terms. The good of the patient is a very complex notion and should be understood taking into account different facts and dimensions. However, in our view, the ethics consultant has the same moral responsibilities as physicians or other health care professionals, i.e. she should act in a way that promotes the wellbeing of the patient both in a medical and a moral sense. What we want to underline is the idea that the consultant does not provide recommendations or advice irrespective of the other parties involved (patient, health professionals, family members and others). The advice offered should be considered the result of a joint moral assessment of the situation, taking into full account the clinical facts, the patient’s moral convictions and life story, and other relevant perspectives. As Sulmasy put it: “[…] the consultant cannot claim a monopoly on moral truth and make the decisions alone. Rather, the consult should facilitate a discussion designed to lead to the best answer, however imperfectly” ([24], p. 102). In this sense, the consultant’s advice is not “absolute”, i.e. *ab-solutus*, that is to say, loosened from the context and the narrative dimension of the given case.

As stated above, the consultant should not be considered an external party detached from the ethical dimension of the case under consideration, but a health care professional involved in patient care. Moreover, even if the “fact” of moral pluralism is acknowledged, this does not relieve us of the task of assessing the acceptability of different moral convictions and perspectives.

## Conclusions

We have argued that the question of neutrality has been a vexing and often implicit question throughout the history of ethics consultation. In particular, it has to do with the fear that consultants may feel tempted to impose their personal beliefs on others and curtail the professional liberty of physicians and other health care providers. This has led ethics consultants to favor less controversial roles and tasks, such as facilitation, mediation, case discussion or analysis. Yet, as we have shown, clinical ethics consultation and clinical care ultimately pursue the same goal and thus have to be guided by the same attitudes. This insight supports an engaged, normatively laden role and responsibility for the ethics consultant. We have distinguished different meanings of the term neutrality with regard to four different elements of the consultation process: the parties, the arguments, the values and norms, as well as the outcome of the consultation process. The practice of making recommendations, which usually is the final step in a consultation, should be seen as a way to help parties to identify the ethically best justified course of action and not as a way to impose a supposed predetermined moral truth, based on the subjective consultant’s point of view. We have proposed a non-authoritarian way to interpret the term “recommendation” as advice, taking a cue from the Greek philosopher Aristotle. The clinical decision makers, the patient, and the patient’s relatives remain free to accept or reject the recommendation or advice as agents responsible for their actions. In fact, the term “advice” already implies an appeal to a person’s reasoning capacities rather than manipulation or coercion to decide or act in a certain way.

## Abbreviation

CEC: Clinical Ethics Consultation
